# Compared to the amniotic membrane, Wharton’s jelly may be a more suitable source of mesenchymal stem cells for cardiovascular tissue engineering and clinical regeneration

**DOI:** 10.1186/s13287-017-0501-x

**Published:** 2017-03-21

**Authors:** Lei Pu, Mingyao Meng, Jian Wu, Jing Zhang, Zongliu Hou, Hui Gao, Hui Xu, Boyu Liu, Weiwei Tang, Lihong Jiang, Yaxiong Li

**Affiliations:** 1 Department of Cardiovascular Surgery, Yan’an Affiliated Hospital of Kunming Medical University, Kunming Medical University, 245, East of Renmin Road, Kunming, 650051 Yunnan People’s Republic of China; 2 Central Laboratory, Yan’an Affiliated Hospital of Kunming Medical University, 245, East of Renmin Road, Kunming, 650051 Yunnan People’s Republic of China; 3 Cardiovascular Surgery Institute of Yunnan, 245, East of Renmin Road, Kunming, 650051 Yunnan People’s Republic of China; 4 Department of Anesthesiology, The Second Affiliated Hospital of Kunming Medical University, 374, Dianmian Road, Kunming, 650051 Yunnan People’s Republic of China; 5 Department of Thoracic Surgery, Yan’an Affiliated Hospital of Kunming Medical University, Kunming Medical University, 245, East of Renmin Road, Kunming, 650051 Yunnan People’s Republic of China; 6 First People’s Hospital of Yunnan Province, 157, Jinbi Road, Kunming, Yunnan People’s Republic of China

**Keywords:** Tissue engineering, Mesenchymal stem cells, Wharton’s jelly, Amniotic membrane, Cardiovascular, Regenerative medicine

## Abstract

**Background:**

The success of developing cardiovascular tissue engineering (CTE) grafts greatly needs a readily available cell substitute for endothelial and interstitial cells. Perinatal annexes have been proposed as a valuable source of mesenchymal stem cells (MSCs) for tissue engineering and regenerative medicine. The objective of the present study is to evaluate the potential of human Wharton’s jelly MSCs (WJ-MSCs) and amniotic membrane MSCs (AM-MSCs) as a seeding cell in CTE and cardiovascular regenerative medicine.

**Methods:**

WJ-MSCs/AM-MSCs were isolated and characterized in vitro according to their morphology, proliferation, self-renewal, phenotype, and multipotency. More importantly, the characteristics of hemocompatibility, extracellular matrix deposition, and gene expression and viability of both MSCs were investigated.

**Results:**

Fibroblast-like human WJ-MSCs and AM-MSCs were successfully isolated and positively expressed the characteristic markers CD73, CD90, and CD105 but were negative for CD34, CD45, and HLA-DR. Both MSCs shared trilineage differentiation toward the adipogenic, osteogenic, and chondrogenic lineages. The proliferative and self-renewal capacity of WJ-MSCs was significantly higher than that of AM-MSCs (*P* < 0.001). WJ-MSCs provided comparable properties of antiplatelet adhesion and did not activate the coagulation cascade to endothelial cells. However, aggregated platelets were visualized on the surface of AM-MSCs-derived cell sheets and the intrinsic pathway was activated. Furthermore, WJ-MSCs have superior properties of collagen deposition and higher viability than AM-MSCs during cell sheet formation.

**Conclusions:**

This study highlights that WJ-MSCs could act as a functional substitute of endothelial and interstitial cells, which could serve as an appealing and practical single-cell source for CTE and regenerative therapy.

## Background

Although surgery and medication have achieved tremendous progress, cardiovascular diseases still represent the leading cause of morbidity and mortality in the developed world [1, 2] and in China. [3] However, the shortage of donor tissues and organs has limited the therapeutic option of end-stage cardiovascular diseases [4]. Therefore, there is an urgent need to develop alternative therapy. In the cardiovascular system, the tissue engineering approach and mesenchymal stem cells (MSCs)-based regenerative medicine are being pursued and increasingly regarded as the promising solution for the development of viable substitutes of heart valves, blood vessels, myocardium, and cardiac patch materials [4–6]. Cardiovascular tissue engineering (CTE) focuses on the regeneration and replacement of diseased cells, tissues, or organs to restore impaired function using the knowledge and techniques of engineering, materials science, cell biology, and molecular biology. For several years, CTE mainly relied on a “classical” scaffold-cell-based paradigm, including the isolation and expansion of seeding cells; subsequently, these cells are seeded onto an appropriate scaffold after in vitro cultivation and finally implantated in vivo. Several challenges still exist in the aim to generate functional cardiovascular grafts and to explore effective cell therapies, searching for an appropriate target cell is one of the primary challenges [7].

For decades, MSCs have been extensively explored as a noncontroversial, multipotent, easily accessible, safely transplantable, and new therapeutic cell source for tissue engineering and regenerative medicine [8, 9]. Tissue engineering and MSCs transplantation have been proposed as the most promising strategies for the regeneration of impaired cardiovascular tissues. As MSCs hold the advantages of homing, multi-differentiation, paracrine, immunomodulation, and anti-inflammation capabilities; they have been widely studied in the clinical arena (http://clinicaltrials.gov/) to alleviate a variety of debilitating disorders [10], such as acute myocardial infarction [11], graft-versus-host disease [12], autoimmune diseases, cystic fibrosis [13], and nerve regeneration, etc. [10, 14]. A number of donor tissue-derived MSCs, including bone marrow, adipose tissue, skin, dental pulp, hair follicle, perinatal tissue, and induced pluripotent stem cells, have been evaluated and their phenotypes, proliferative potential, multipotent differentiation potential, and potent immunomodulatory properties identified [9, 10, 15, 16]. However, the therapeutic application of adult tissue-derived MSCs, such as bone marrow and adipose, may be limited by aging and diseases by decreasing their quality and quantity [10, 17]. Furthermore, obtaining autologous MSCs undergoes the disadvantages of invasive isolation procedure, which would undermine the integrity of local tissue and would be accompanied with significant risks [17, 18]. Therefore, the clinical applications of an alternative MSCs source has to be further explored.

Perinatal annexes are seen as a biological waste post-delivery and represent a reservoir of MSCs, as clinical-scale cell numbers can be easily attained from several perinatal annexes, such as umbilical cord blood, Wharton’s jelly (the gelatinous matrix in the umbilical cord), placental tissue, and amniotic membrane [18, 19]. MSCs isolated from perinatal annexes are ideal substitutes, as they can overcome the limitations and disadvantages of adult MSCs as mentioned earlier. These MSCs are maintained in the embryological phase and represent a bridge between embryonic and adult stem cells. As primitive MSCs, Wharton’s jelly MSCs (WJ-MSCs) and amniotic membrane MSCs (AM-MSCs) have been successfully isolated and identified as homogeneous MSCs in Wharton’s jelly and amniotic membrane, and they can be induced to differentiate into osteoblasts, adipocytes, and chondrocytes. Moreover, WJ-MSCs have been successfully induced to differentiate into cardiomyocytes and endothelial-like cells at certain conditions [20, 21]. WJ-MSCs are easily accessible and allow for cell harvest from the donor without substantial risks and sacrifice of intact donor tissues [22]. WJ-MSCs also can be transplanted into the same donor and allow allogenic transplantation without ethical and immunological barriers; because it is an immunologically privileged status, a single allogeneic WJ-MSCs donor may serve for substantial patients. These advantages make WJ-MSCs and AM-MSCs a fascinating source of stem cells for CTE and could overcome the challenge of using mature somatic cells from adult tissues. Thus, it is interesting to compare the application potential of WJ-MSCs and AM-MSCs for CTE and regenerative medicine.

To fulfill its roles in CTE, seeding cells must functionally replace resident endothelial cells (EC) with antithrombogenic properties and terminally differentiated stromal cells responsible for extracellular matrix (ECM) development [23, 24]. Considering the observed efficiency of WJ-MSCs/AM-MSCs and the previous studies of WJ-MSCs/AM-MSCs-based cardiovascular regenerative medicine and tissue engineering [25–28], it is surprising that so little knowledge is known about the head-to-head comparison of the properties of antiplatelet adhesion, hemocompatibility, and the profiles of ECM deposition and cell viability of WJ-MSCs/AM-MSCs. Consequently, the motivation of the present study is to compare the applications of WJ-MSCs/AM-MSCs for CTE on hemocompatibility and ECM secretion. Results demonstrated that WJ-MSCs are superior seeding cells for developing tissue-engineered cardiovascular grafts.

## Methods

### Isolation and culture of MSCs

Primary human WJ-MSCs/AM-MSCs (*n* = 5, gestational age 38–40 weeks) were isolated from human umbilical cord Wharton’s jelly and amniotic membrane as previously described [29]. Briefly, the umbilical cord and amniotic membrane were obtained from a healthy baby and rinsed thoroughly with phosphate-buffered saline (PBS). The cord was maintained in PBS at 4 °C and delivered to the laboratory and processed immediately. After the umbilical cord arteries and vein were dissected, the adventitia of umbilical cord was gently scraped using a scalpel blade. The Wharton’s jelly and the amniotic membrane were cut into small cubes and placed in tissue culture dishes (Corning, Lowell, MA, USA) in a sterile fashion. Explants were cultured in α-minimum essential medium (α-MEM; Hyclone, Logan, UT, USA) supplemented with 20% fetal bovine serum (FBS; HyClone) and antibiotics (1% penicillin/streptomycin). Subsequently, the culture dish was maintained at 37 °C in a humidity incubator containing 5% CO_2_. The culture medium was changed every third day until MSCs migrated onto the culture dish. After 3 to 4 weeks, WJ-MSCs/AM-MSCs were detached and passaged using 0.0625% trypsin while cells were propagated to reach 70 to 90% confluency. In these experiments, cells from the third to fifth passages were used and maintained at 37 °C in a humidified atmosphere containing 5% CO_2_.

### Cell metabolism and proliferation

To investigate the cell metabolism and proliferation potential, the 3-(4, 5-dimethylthiazol-2-yl)-2,5-diphenyltetrazolium bromide (MTT) colorimetric assay was used. Briefly, WJ-MSCs and AM-MSCs suspensions were diluted to 2 × 10^3^ cells ml^-1^ with α-MEM containing 10% FBS. Then, 100 μL aliquots of WJ-MSCs and AM-MSCs suspensions were seeded into 96-well plates (Corning) and incubated at 37 °C in a humidified atmosphere with 5% CO_2_. After allowing 24 h for cell adherence and proliferation, five wells of each group were used for the MTT assay, which were carried out each day at the same time of a 7-day culture period. A total volume of 20 μL of 5 mg mL^-1^ MTT (Sigma-Aldrich, St. Louis, MO, USA) solution was added to each well and incubated for 4 h, and then the medium was discarded. Subsequently, 150 μL dimethylsulfoxide (DMSO; MP Biomedicals, Santa Ana, CA, USA) was added to dissolve formazan salts, and the absorbance values for each well were measured using a spectrophotometer at 490 nm (Model 680; Bio-Rad, Hercules, CA, USA).

### Colony-forming unit fibroblast (CFU-F) assay

The self-renewal capacity of WJ-MSCs and AM-MSCs was evaluated using the CFU-F assay. A total of 1 × 10^3^ of WJ-MSCs or AM-MSCs (P4) were suspended in basal cultivation medium and cultured in 100-mm-diameter culture plates (Corning). These cells were washed on day 14 with PBS and stained with 0.3% crystal violet (Sigma-Aldrich) for 8 min at room temperature. Subsequently, the stained cultures were photographed and analyzed by using inverted microscopy, and the colonies with 50 or more cells were counted.

### Immunophenotype analysis by flow cytometry

For the analysis of the immunophenotype and quantification of antigen expression, flow cytometry was performed by using antibodies against CD29, CD73, CD90, CD105, CD146, α-smooth muscle actin (α-SMA), CD34, CD45, and HLA-DR. Briefly, 6 × 10^5^ WJ-MSCs/AM-MSCs were washed with PBS three times and incubated with fluorescein isothiocyanate (FITC)-CD90, CD105, HLA-DR, α-SMA and phycoerythrin (PE)-CD73, CD34 and peridinin-chlorophyll protein (Percp)-CD29, and CD45-conjugated mouse anti-human monoclonal antibodies (BD Biosciences, San Jose, CA, USA) for 30 min at room temperature in the dark. Isotype-matched IgGs served as the control. Cells were subjected to flow cytometric analysis using a Beckman Coulter Epics XL instrument (Beckman Coulter, Fullerton, CA, USA).

### Multi-lineage differentiation

The multi-lineage differentiation potential of WJ-MSCs and AM-MSCs were determined by using the inducing differentiation reagents, MSC go Adipogenic, MSC go Osteogenic, and MSC go Chondrogenic (Biological Industries, Kibbutz Beit-Haemek, Israel), according to the manual’s instructions. Briefly, 1 × 10^5^ WJ-MSCs or AM-MSCs (P4) were cultured with a-MEM in six-well plates without the addition of inducing regents until 80 to 90% confluence. Then, the culture medium was changed to adipogenic or osteogenic differentiation medium. α-MEM supplied with 2% FBS served as the negative control. The induction medium was refreshed at 3-day intervals. For adipogenic identification, cells were fixed with 10% formaldehyde after 4 weeks of culture and stained with 0.3% Oil Red O (Sigma-Aldrich), and lipid droplets were identified microscopically. For osteogenic identification, cells were fixed with 4% paraformaldehyde after 3 weeks of culture and stained with 2% Alizarin Red S (pH 4.2; Kermel, Tianjin, China), and the deposited calcification nodules were identified microscopically.

For chondrogenic differentiation, a total of 5.5 × 10^5^ WJ-MSCs or AM-MSCs suspensions were centrifuged at 250 *g* for 5 min for obtaining cell pellets. After draining the supernatant carefully, 1 ml of MSC go Chondrogenic differentiation medium was added. The induction medium was refreshed at 4-day intervals. α-MEM supplied with 2% FBS served as the negative control. After 3 weeks of cultivation, cells were fixed with 10% formaldehyde for 24 h and embedded in paraffin. Sections (4 μm) were deparaffinized in xylene and stained with Alcian Blue Staining Kit (ScienCell, Carlsbad, CA, USA) according to the user’s manual. Then, the morphology of cartilage lacuna and sulfated proteoglycan were identified.

### Evaluation of platelet adhesion

Platelet adhesion was evaluated by incubating platelet-rich plasma (PRP) with WJ-MSCs, AM-MSCs and human umbilical vein endothelial cells (HUVECs) in 24-well plates with one coverslip (tissue culture-treated; 8 mm) well^-1^. Non-cell-seeded wells were served as the control. WJ-MSCs and AM-MSCs were grown in α-MEM supplemented with 10% FBS and 1% penicillin/streptomycin. HUVECs were provided by the Central Laboratory of Yan’an Affiliated Hospital of Kunming Medical University and cultured with EC growth medium (Medium 200; Gibco, Grand Island, NY, USA) supplemented with 2% FBS, epidermal growth factor (EGF) 5 ng ml^-1^, basic fibroblast growth factor (bFGF) 3 ng ml^-1^, heparin 10 μg ml^-1^, bovine serum albumin (BSA) 200 ng ml^-1^, hydrocortisone 1 ng ml^-1^, gentamicin 0.5 mg ml^-1^, and amphotericin B (25 μg ml^-1^). WJ-MSCs, AM-MSCs, and HUVECs were passaged by trypsinization (0.0625% trypsin/EDTA) until 90% confluence and subcultured in 24-well plates at a density of 10,000 cells cm^-2^.

To obtain PRP, whole blood from a healthy adult volunteer, free of medication, was drawn into a glass syringe containing 3.8% sodium citrate (blood/sodium citrate volume, 9:1), with informed consent. PRP was acquired by centrifugation of the whole blood at 200 *g* for 10 min at 22 °C. After cell culture medium was drained and rinsed two times with PBS, PRP was gently pipetted onto cells in each well (200 μl well^-1^) and incubated for 30 min at 37 °C. Then, PRP was drained into the original syringe and platelet counts were performed using an automated routine blood analyzer (Sysmex XT-4000i; Sysmex, Kobe, Japan). The plates were rinsed three times with PBS (5 min each) with gentle agitation to eliminate the weakly adhered platelets and then fixed in 4% glutaraldehyde for 24 h. Subsequently, the samples were washed in PBS and dehydrated in a series of ethanol solutions. Then subjected to critical-point drying and sputter-coated with gold, the platelets that attached to each surface were observed using a Hitachi S-3000 N Scanning Electron Microscope (SEM; Hitachi, Tokyo, Japan).

### Hemocompatibility

More importantly, the hemocompatibility of WJ-MSCs and AM-MSCs were investigated by the measurements of prothrombin time (PT) and activated partial thromboplastin time (APTT). Similar to platelet adhesion assessment, whole blood was added to 24-well plates (1 ml well^-1^) and incubated for 30 min at 37 °C. Then, the blood was drained into a novel tube and centrifuged at 250 *g* for 10 min at 22 °C. PT and APTT were measured using an automated blood coagulation analyzer (Sysmex CS-5100). Control experiments were carried out using HUVECs and normal blood sample. Each experiment was repeated three times.

### Preparation of cell sheet

The cryopreserved WJ-MSCs and AM-MSCs (P4) were rapidly thawed and cultivated in α-MEM supplied with 10% FBS. At 90% confluence, cells were trypsinized and seeded in a six-well plate (Corning) with a density of 1.0 × 10^5^ cells cm^-2^ and cultured in α-MEM supplied with 10% FBS, ascorbic acid (50 μg ml^-1^, Sigma-Aldrich), and 1% penicillin/streptomycin. Cells were incubated in a humidified atmosphere of 5% CO_2_, at 37 °C and formed a cohesive living cell sheet. Normal mouse thoracic aorta smooth muscle cell (SMC), A7r5 cell line (mSMC-A7r5; Cell Bank of Kunming Institute of Zoology, Chinese Academy of Sciences), served as the positive control. mSMC-A7r5 was cultivated in high-glucose Dulbecco’s Modified Eagle’s Medium (DMEM; Gibco) at the same cell-seeding density and conditions. After 12 days of preparation, inverted microscopic observations were performed. The intact cell sheets of WJ-MSCs and AM-MSCs were harvested from the plate by using a cell scraper.

### Histological assessment

Cell sheet samples were fixed for 24 h in 10% formaldehyde at room temperature followed by dehydration and embedding in paraffin. Cross-sections (4 μm) were cut on a microtome and mounted on glass slides. The general morphology and structure were evaluated via standard hematoxylin and eosin (H&E) staining. The ECM of elastin laminae, collagen, and glycosaminoglycan was investigated using Movat’s pentachrome staining according to the manufacturer’s instructions (ScyTek Laboratories, Logan, UT, USA). The H&E and Movat’s pentachrome staining results were evaluated using a Nikon DS-Ri1 compound microscope (Nikon, Tokyo, Japan).

### Electron microscope

Cell sheet samples were prepared for SEM and transmission electron microscopy (TEM) by fixation and dehydration. Briefly, cell sheets were washed with PBS and fixed with 3.5% glutaraldehyde (Alfa Aesar, Ward Hill, MA, USA) for 24 h at 4 °C. The samples were postfixed and processed in 1% OsO_4_ and dehydrated in standard graduated ethanol series. After being critical-point dried and sputter-coated with gold, the samples were imaged using a Hitachi S-3000 N SEM.

For TEM investigation, postfixation samples were embedded in epoxy resin. Ultra-thin 60 nm sections were cut and investigated using a JEOL 1011 TEM (Jeol, Zaventem, Belgium) at 100 kV.

### Quantification of ECM deposition

To evaluate the ECM secretion capacity of WJ-MSCs and AM-MSCs during cell sheet preparation, Sircol™ soluble collagen assay and Fastin™ elastin assay (Biocolor, Carrickfergus, UK) were performed.

To evaluate the collagen content of the acquired cell sheet, the collagens were quantified using Sircol™ soluble collagen assay according to the manufacturer’s instructions. Briefly, collagen was extracted by using acid-pepsin solution (0.1 mg ml^-1^) with overnight incubation at 4 °C. The supernatant was decanted off into a capped conical microcentrifuge tube. Subsequently, the solution was incubated at 0 °C overnight for isolation and concentration. Then, collagen was dyed with Sirius Red. After washing and draining, the absorbance of the dissolved collagen-bound dye was measured at 570 nm and expressed as micrograms per well of six-well plate.

For investigation the elastin contents of the acquired cell sheet, the Fastin™ elastin assay was used. Briefly, elastin was isolated using 0.25 mol L^-1^ oxalic acid incubated in a 100 °C water bath for 60 min of three cycles, then, elastin was precipitated and dyed. After recovery of elastin-bound dye, the absorbance was measured at 490 nm and expressed as micrograms per well of six-well plate.

### RNA isolation and real-time polymerase chain reaction (real-time PCR)

Total RNA was extracted from WJ-MSCs- and AM-MSCs-based cell sheets in each group and HUVECs by using TRIzol reagent (Molecular Research Center, Cincinnati, OH, USA). Approximately 2 to 5 μg total RNA were converted to cDNA using the SuperScript First-Strand Synthesis Kit (Thermo Fisher Scientific, Waltham, MA, USA). Real-time PCRs were performed using the QuantiTect SYBR Green PCR Kit (Toyobo, Osaka, Japan) and the Applied Biosystems 7500 Real-time PCR Detection System (Applied Biosystems, Foster City, CA, USA). Two independent experiments were performed for each reaction, in triplicate. The expression level of each gene was normalized to the expression of β-actin. A portion of primer sets were purchased from GeneCopoeia and the other primer sets used in this experiment are listed in Table 1. Genes and catalogue numbers of the purchased primer sets were presented as follows: fibronectin, HQP006028; laminin, HQP008000; nidogen, HQP011835; heparin sulfate proteoglycan (HSPG), HQP009107; nitric oxide synthase (NOS), HQP088415.

**Table 1:** Primers sequences for gene expression analysis using real-time PCR

Genes	Accession number	Primer sequence (5′-3′)	Product size (bp)
Collagen-I	NM_000088.3	Forward: AAGGTGTTGTGCGATGACG Reverse: TGGTCGGTGGGTGACTCTG	116
Collagen-IV	NM_001303110.1	Forward: AGGAGACTTCGCCACCAA Reverse: GGTCCTGTGCCTATAACAATT	264
Vimentin	NM_003380.3	Forward: CTGAACCTGAGGGAAACTAA Reverse: AGAAAGGCACTTGAAAGCT	231
Connexin-43	NM_000165.4	Forward: CAGTCTGCCTTTCGTTGT Reverse: CTCTTCCTTTCGCATCAC	168
Vitronectin	NM_000638.3	Forward: CAAAGGCTACCGTTCACAA Reverse: AGACACTCTGGATGGGTTCA	182
Elastin	NM_001278918.1	Forward: CGCTGGTGCTCTTATCTTC Reverse: GCTTCAGGTGCTTGGGTA	176
β-Actin	NM_005559.3	Forward: AGCGAGCATCCCCCAAAGTT Reverse: GGGCACGAAGGCTCATCATT	285

### Live/dead assays for cell viability

After cell sheet preparation, the cell viability of each sheets was characterized using fluorescence staining with Live/Dead assay kit (Merck Millipore, Darmstadt, German). Briefly, cell sheets were rinsed three times with PBS and then incubated in a Live/Dead staining solution according to the manufacturer’s instructions. Then, the submerged cell sheets were incubated at 37 °C for 60 min, washed with PBS, and examined with a Nikon DS-Ri1 fluorescence microscope. The live cells were visualized by green fluorescein and dead cells were represented as red.

### Statistical analysis

Unless otherwise noted, all experiments were performed at least in triplicate (*n* = 5), and representative images were reported. Quantitative experimental results were expressed as the means ± SD. Statistical comparisons were performed using unpaired sample *t* tests for two groups and one-way analysis of variance (ANOVA) and Tukey’s post hoc test for more than two groups. Statistical significance was accepted at *P* < 0.05.

## Results

### Culture and characterization of WJ-MSCs and AM-MSCs

**Fig. 1:**
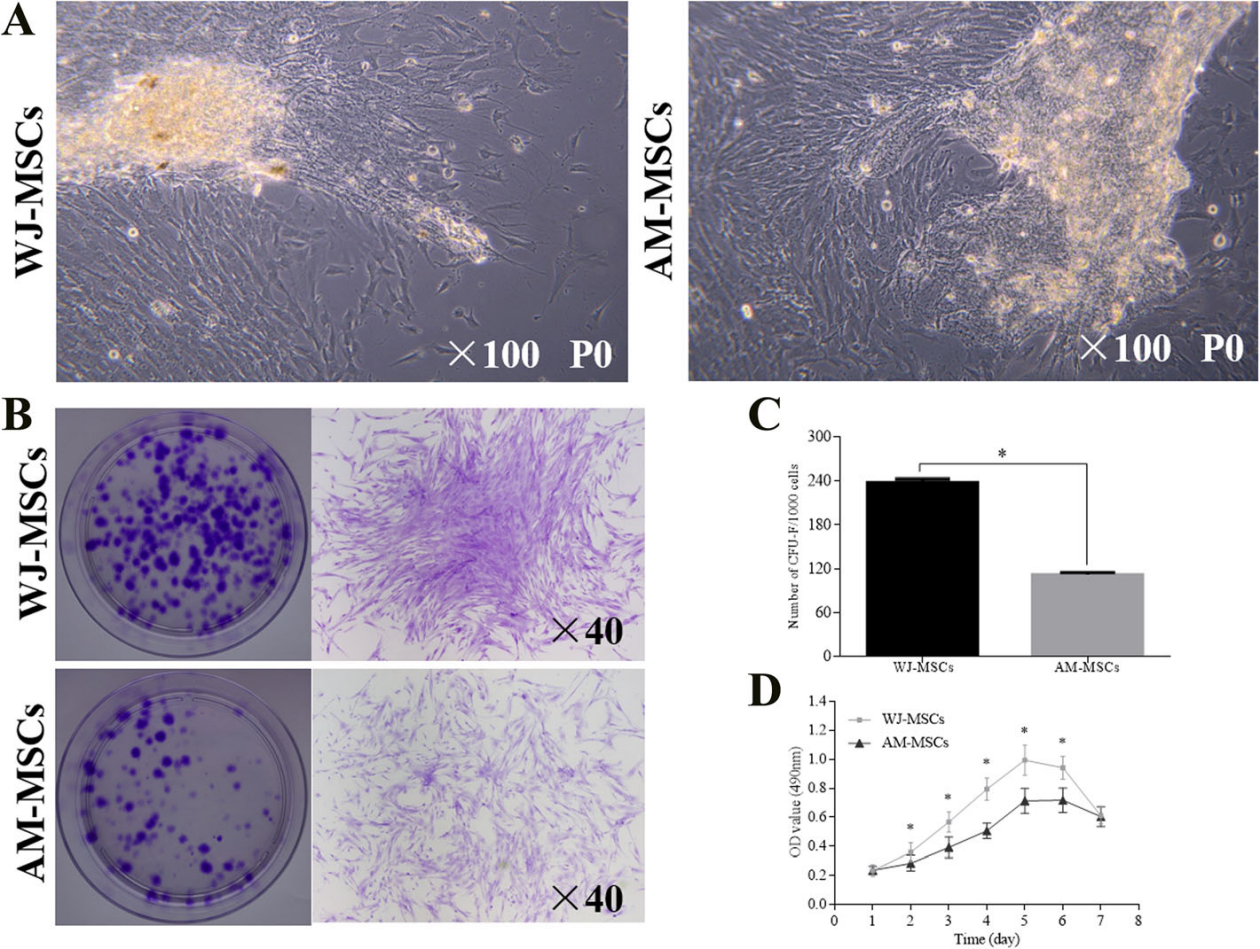
Isolation and characterization of clonogenic and proliferative potential of WJ-MSCs and AM-MSCs. **a** Morphologies of WJ-MSCs and AM-MSCs observed under inverted microscopy. **b** Representative images showing the colony number and a single clone of CFU-Fs observed from WJ-MSCs and AM-MSCs. **c** Cumulative CFU-F numbers of WJ-MSCs and AM-MSCs. **d** Proliferation and metabolism of WJ-MSCs and AM-MSCs as investigated by the MTT assay. (**c** and **d**) Mean ± SD of five independent donors. ^*^ *P* < 0.001, significant difference between the two cell sources (CFU-F and MTT assays at the same time point). *AM-MSCs* amniotic membrane mesenchymal stem cells, *CFU-F* colony-forming unit fibroblast, *WJ-MSCs* Wharton’s jelly mesenchymal stem cells

Primary WJ-MSCs and AM-MSCs were successfully isolated from human umbilical cord Wharton’s jelly and amniotic membrane from all five donors and passaged in vitro on a standard plastic surface. Both WJ-MSCs and AM-MSC (Fig. 1a) exhibited long spindle-like structures and displayed a typical fibroblastic morphology. An important issue of interest in seeding cells for tissue engineering is the self-renewal and proliferative potential. The obtained MSCs were characterized in terms of the CFU-F assay. Both types of cells displayed colony-forming ability, and high colony-forming efficiencies were observed in WJ-MSCs compared to AM-MSCs (Fig. 1b). The number of CFU-F per 100 WJ-MSCs (23.7% ± 4.5%) was significantly higher than that of AM-MSCs (11.1% ± 3.0%; *P* < 0.001; Fig. 1c). The proliferation and metabolic activity of WJ-MSCs were significantly higher than that of AM-MSCs after 2 days of incubation (*P* < 0.001), although they exhibited a similar metabolic activity on the seventh day (Fig. 1d).

**Fig. 2:**
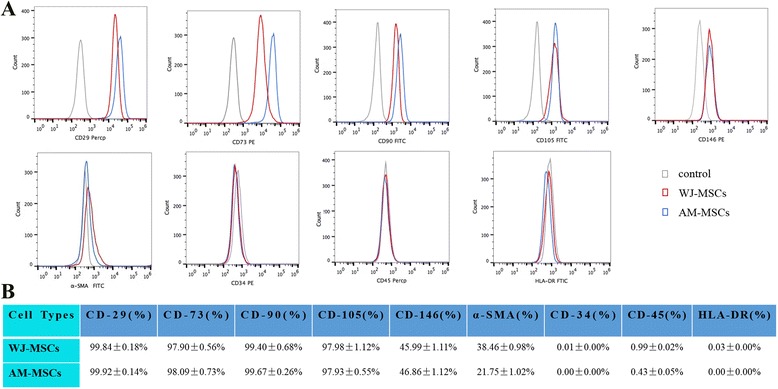
Identification of the immunophenotype of WJ-MSCs and AM-MSCs at P3 by flow cytometric analysis. **a** Representative result from five independent experiments. **b** Percentage of surface marker expression of WJ-MSCs and AM-MSCs. Mean ± SD of five independent experiments. *AM-MSCs* amniotic membrane mesenchymal stem cells, *WJ-MSCs* Wharton’s jelly mesenchymal stem cells

Flow cytometric assay was performed with MSCs derived from Wharton’s jelly and amniotic membrane tissues. Results revealed a typical pattern for MSCs [positive expression of CD29, CD73, CD90, CD105; partial expression of CD146, α-SMA (a typical SMC marker); and negative expression of CD34, CD45, and HLA-DR], indicating that WJ-MSCs and AM-MSCs were representative of a MSCs-like population (Fig. 2).

**Fig. 3:**
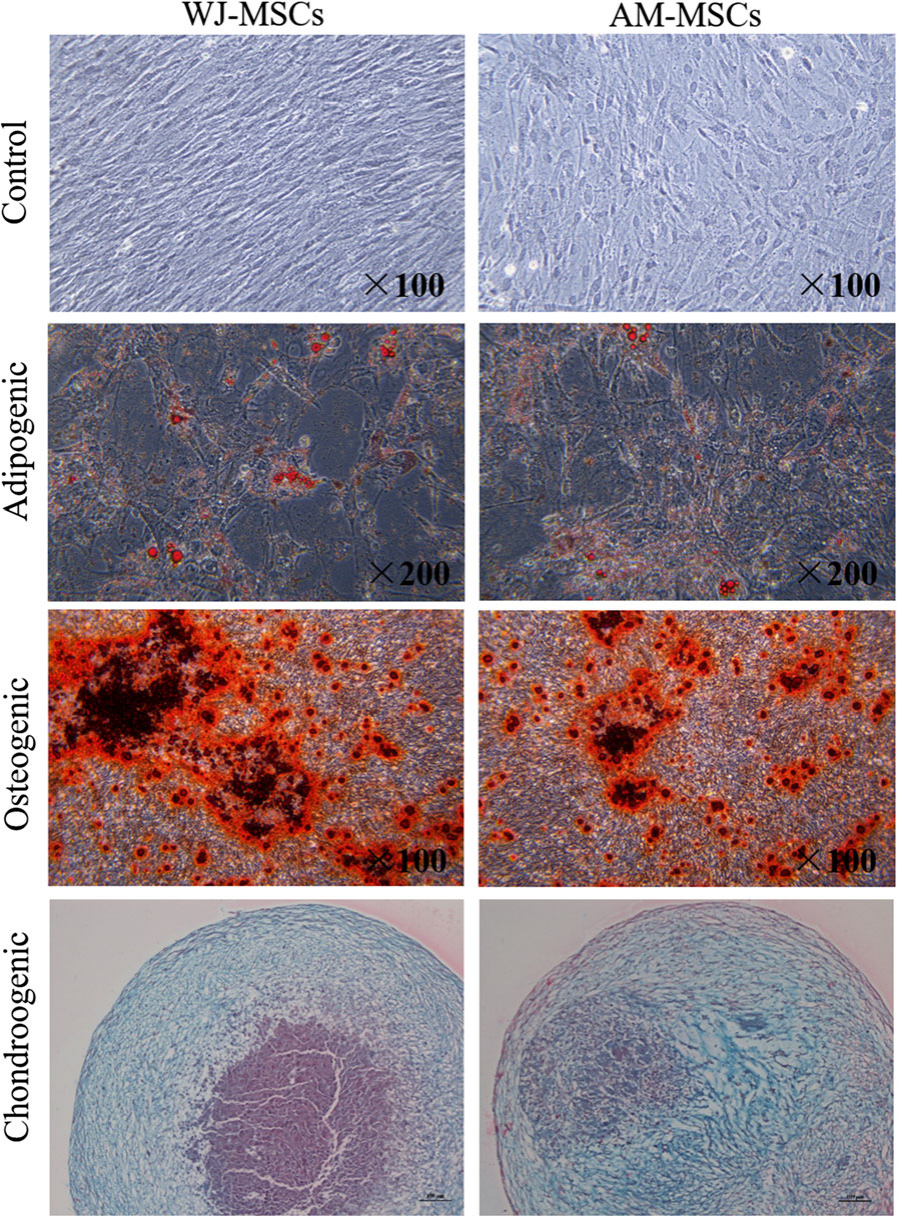
Representative images of multi-lineage differentiation potential of isolated WJ-MSCs and AM-MSCs. WJ-MSCs and AM-MSCs were induced to differentiate toward adipogenic lineage and verified by Alizarin *Red* S staining (magnification, ×100), osteogenic lineage and verified by Oil *Red* O staining (magnification, ×200), and chondrogenic lineage and verified by Alcian *Blue* staining (magnification, ×200). Scale bar, 100 μm. *AM-MSCs* amniotic membrane mesenchymal stem cells, *WJ-MSCs* Wharton’s jelly mesenchymal stem cells

When WJ-MSCs and AM-MSCs were cultured in conditional induction medium, cells were subjected to adipogenic, osteogenic and chondrogenic differentiation (Fig. 3). Adipogenic differentiation was verified by the accumulation of cytoplasmic lipid vacuoles and Oil Red O staining. Osteogenic differentiation was revealed by Alizarin Red S-positive mineral deposits, which indicated the WJ-MSCs’ and AM-MSCs’ osteogenic potential. Moreover, chondrogenic differentiation was evaluated by Alcian Blue staining with ECM, such as collagen and glycosaminoglycans. These results suggested that WJ-MSCs and AM-MSCs were successfully isolated from the human umbilical cord Wharton’s jelly and amniotic membrane tissues. Furthermore, WJ-MSCs are a primitive stem cell population with higher application potential.

### Platelet adhesion and hemocompatibility of WJ-MSCs and AM-MSCs

**Fig. 4:**
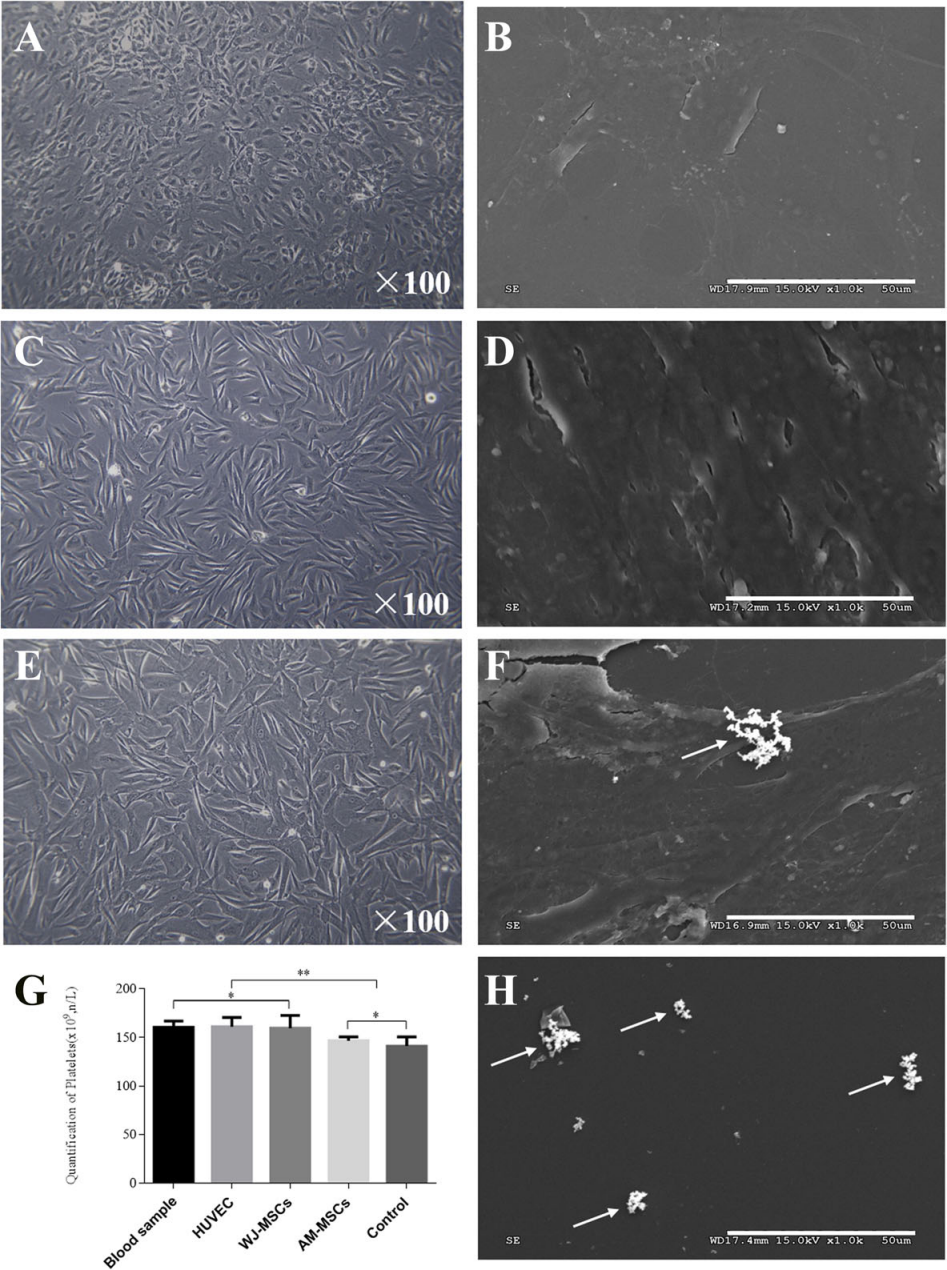
Antiplatelet adhesion properties of WJ-MSCs and AM-MSCs. Morphologies of HUVECs (**a**), WJ-MSCs (**c**), and AM-MSCs (**e**). Representative SEM images of platelets adhered, HUVECs (**b**), WJ-MSCs (**d**), AM-MSCs (**f**), and non-cell-seeded control group (**h**). *White arrow* indicates the aggregated platelets. (**g**) Quantitative evaluation of nonadherent platelets in plasma. Mean ± SD. ^*^ *P* > 0.05, no statistical significance between groups; ^**^ *P* < 0.05, significant difference between groups. *AM-MSCs* amniotic membrane mesenchymal stem cells, *HUVECs* human umbilical vein endothelial cells, *WJ-MSCs* Wharton’s jelly mesenchymal stem cells

A dogma in the construction of CTE substitutes is to seed EC as a confluent surface to prevent thrombosis. To investigate the antithrombogenic properties of WJ-MSCs and AM-MSCs, HUVECs-seeded (negative) and non-cell-seeded (positive) cultivation plate surfaces served as the controls and healthy donor blood was used. As expected, adhered and aggregated platelets were obviously observed on the non-cell-seeded plate surface (Fig. 4h), which represented coral-like structures. WJ-MSCs (Fig. 4d) and HUVECs (Fig. 4b) represented similar antiplatelet adhesion properties; however, platelet adhesion and aggregation was observed on the AM-MSCs-seeded surface (Fig. 4f). Quantitative analysis of platelets in the plasma also demonstrated significantly higher platelet number of HUVECs and WJ-MSCs than that of AM-MSCs and non-cell-seeded surface (*P* < 0.05; Fig. 4g). There was no significant difference between blood sample, HUVECs, and WJ-MSCs (*P* > 0.05). Moreover, there was no significant difference between AM-MSCs and non-cell-seeded surface (*P* > 0.05). These results revealed that WJ-MSCs had antiplatelet adhesion properties similar to HUVECs.

**Fig. 5:**
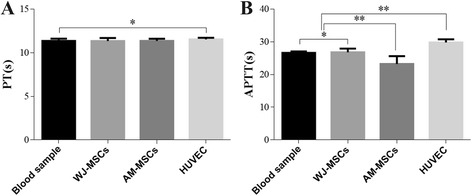
PT and APTT of different cells and normal blood samples. Mean ± SD of five independent experiments performed in triplicate. ^*^ *P* > 0.05; ^**^ *P* < 0.05; ^***^ *P* < 0.001. *APTT* activated partial thromboplastin time, *AM-MSCs* amniotic membrane mesenchymal stem cells, *HUVECs* human umbilical vein endothelial cells, *PT* prothrombin time, *WJ-MSCs* Wharton’s jelly mesenchymal stem cells

Results of the hemocompatibility assessment of WJ-MSCs, AM-MSCs, and HUVECs are presented in Fig. 5. As expected, the extrinsic (PT) and intrinsic (APTT) pathways of the coagulation cascade were not activated with WJ-MSCs; however, the intrinsic pathway was activated with AM-MSCs. There was no significant difference of PT in groups (Fig. 5a) and the APTT of the WJ-MSCs was approximated to blood sample (*P* > 0.05). However, the APTT of the HUVECs was prolonged and the AM-MSCs was shortened (*P* < 0.05). These data strongly suggest that WJ-MSCs can be used as an alternative seeding cell to EC in CTE.

### Histology of cell sheet

**Fig. 6:**
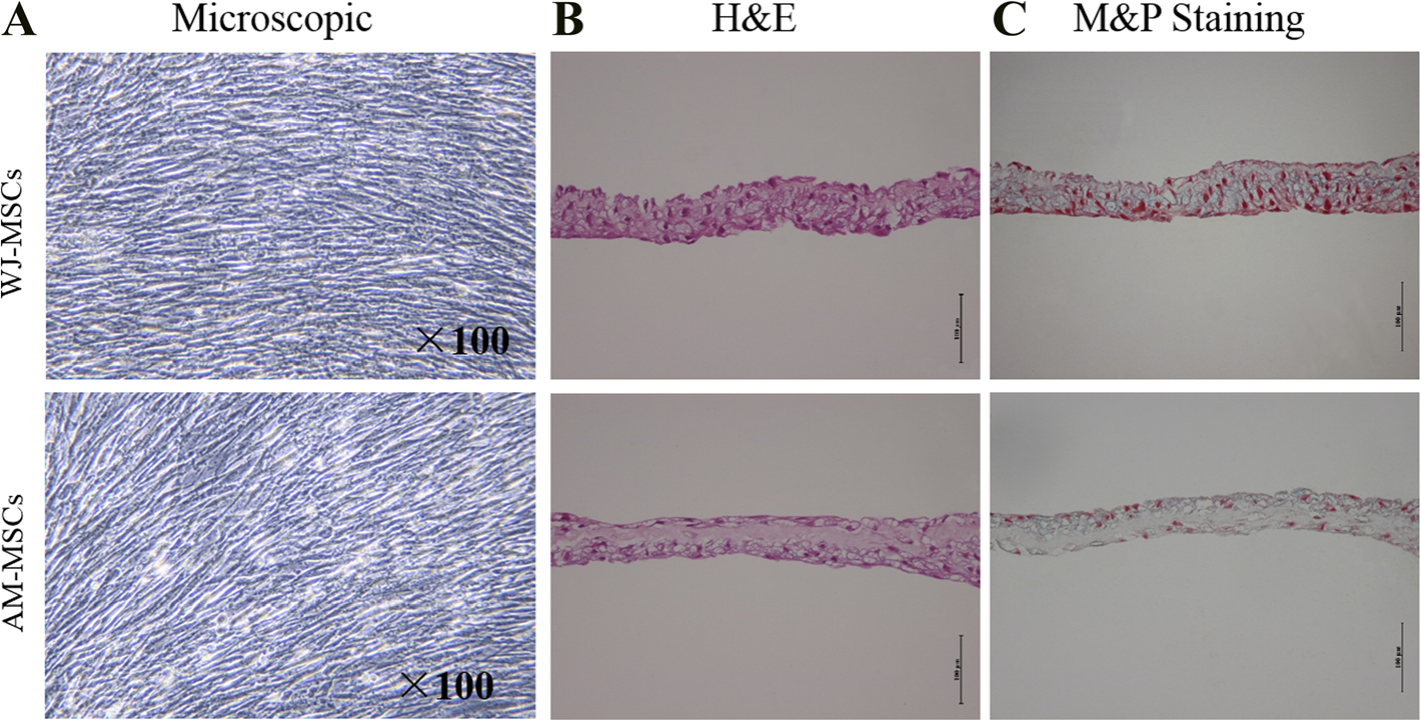
Representative microscopic images and histology evaluation of WJ-MSC- and AM-MSC-derived cell sheets. **a** Acquired cell sheet imaged by an inverted microscope on day 12 (magnification, ×100). **b** H&E staining of WJ-MSC- and AM-MSC-derived cell sheets. Scale bar, 100 μm. **c** Movat’s pentachrome staining of WJ-MSC- and AM-MSC-derived cell sheets. Scale bar, 100 μm. *AM-MSCs* amniotic membrane mesenchymal stem cells, *WJ-MSCs* Wharton’s jelly mesenchymal stem cells

To be effective in practical applications of seeding cells for CTE, ECM must be produced and organized by seeding cells. To evaluate cell sheet assembly and ECM deposition, WJ-MSCs, AM-MSCs, and mSMA-A7r5 were cultured in basic medium supplied with 10% FBS and ascorbic acid (50 μg/ml). Results showed that both types of MSCs deposit a certain amount of ECM and formation of cell sheet; however, the cell sheet was not successfully generated of mSMA-A7r5. MSCs-derived sheets were observed with inverted microscope and demonstrated confluent cell connection (Fig. 6a).

H&E staining of cross-sectional samples revealed that both WJ-MSCs and AM-MSCs sheets were four to five layers, and ECM spread uniformly as a three-dimensional structure. However, more stained nuclei were visualized in the WJ-MSCs sheets (Fig. 6b). Next, fibrous ECM structure and deposition of the cell sheet samples were analyzed by Movat’s pentachrome staining. As expected, the presence of a dense extracellular matrix rich in proteoglygcans (blue) in which cells were embedded; nevertheless, there was no obviously stained collagen (yellow) and elastin (brown; Fig. 6c). The ECM was homogeneous and did not present any significant discrepancy between them.

### Electron microscope

**Fig. 7:**
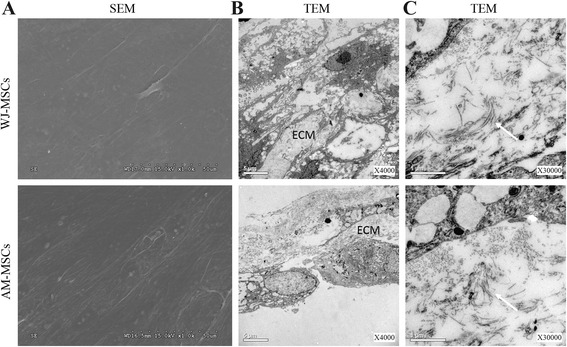
Representative images of electron microscopic analysis of WJ-MSC- and AM-MSC-derived cell sheets. (**a**) SEM images of the surface of both cell sheets. Scale bar, 50 μm. (**b** and **c**) TEM images of the ultrastructure of the ECM and collagen fibers (*white arrow*) within both cell sheets. Scale bar, 5 μm (**b**) and 1 μm (**c**). *AM-MSCs* amniotic membrane mesenchymal stem cells, *ECM* extracellular matrix, *SEM* scanning electron microscope, *TEM* transmission electron microscopy, *WJ-MSCs* Wharton’s jelly mesenchymal stem cells

As expected, SEM showed the surface morphology of both WJ-MSCs and AM-MSCs sheets were spread and elongated, and a dense film-like confluent cell network was formed and retained tight cell junctions (Fig. 7a). Subsequently, the intracellular ultrastructures of both cell sheets were examined by TEM (Fig. 7b and c). The data indicated that the acquired cell sheets preserved the intercellular junctions and endogenous ECM. Collagen fibrils were present at a high density in the cell sheets. The collagen fibers concentrated into clusters and were distributed in random directions throughout the cell sheets.

### ECM quantification and real-time PCR

**Fig. 8:**
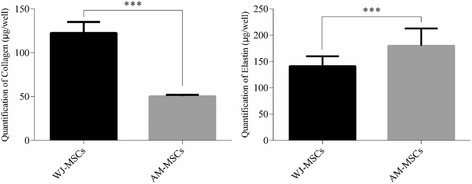
Quantitative analysis of collagen and elastin of WJ-MSC- and AM-MSC-derived cell sheets. Mean ± SD of five independent experiments performed in triplicate. ^***^ *P* < 0.001. *AM-MSCs* amniotic membrane mesenchymal stem cells, *WJ-MSCs* Wharton’s jelly mesenchymal stem cells

Histological and electron microscope results showed that ECM was deposited by WJ-MSCs and AM-MSCs during the process of cell sheet formation. However, the ECM secretion ability of both MSCs was still unknown. The secretion of the major ECM proteins, such as collagen and elastin were determined by using Sircol™ soluble collagen assay and Fastin™ elastin assay (Fig. 8). Results suggested that WJ-MSCs had a more preferable nature of collagen secretion than AM-MSCs (*P* < 0.001). On the contrary, AM-MSCs had a significant property of elastin deposition than WJ-MSCs (*P* < 0.001).

**Fig. 9:**
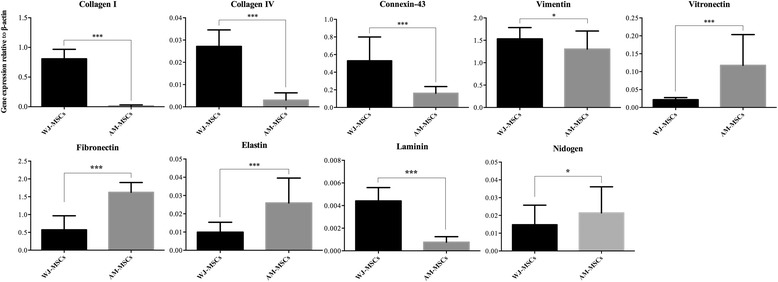
Expression of ECM-related genes, including collagen I, collagen IV, connexin-43, vimentin, vitronectin, and elastin of both WJ-MSC- and AM-MSC derived cell sheets as determined by real-time PCR. The expression levels were normalized to that of β-actin. Mean ± SD of five independent experiments performed in triplicate. ^*^ *P* > 0.05; ^***^ *P* < 0.001. *AM-MSCs* amniotic membrane mesenchymal stem cells, *WJ-MSCs* Wharton’s jelly mesenchymal stem cells

To further evaluate the transcript expression levels of selected ECM genes (including collagen I, collagen IV, connexin-43, vimentin, vitronectin, fibronectin and elastin) and defined basement membrane genes (laminin and nidogen) of both WJ-MSCs- and AM-MSCs-derived cell sheets, real-time PCR was performed. Results are presented in Fig. 9. Data revealed that the expression of collagen I, collagen IV, connexin-43, and laminin of WJ-MSCs sheets was significantly higher than that of AM-MSCs sheets (*P* < 0.001). However, the expression of vitronectin, fibronectin, and elastin of the WJ-MSCs sheets were significantly lower than that of the AM-MSCs sheets (*P* < 0.001). There was no significant statistical difference of vimentin and nidogen between the WJ-MSCs and AM-MSCs sheets (*P* > 0.05).

**Fig. 10:**
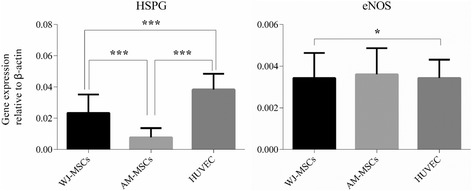
Expression of HSPG and NOS of both WJ-MSC- and AM-MSC-derived cell sheets as well as HUVECs. Mean ± SD of five independent experiments performed in triplicate. ^*^ *P* > 0.05; ^***^ *P* < 0.001. *AM-MSCs* amniotic membrane mesenchymal stem cells, *eNOS* endothelial nitric oxide synthase, *HSPG* heparin sulfate proteoglycan, *HUVECs* human umbilical vein endothelial cells, *WJ-MSCs* Wharton’s jelly mesenchymal stem cells

To elucidate the underlying mechanism of the antiplatelet adhesion properties and the nonactivation of the intrinsic coagulation pathway of WJ-MSCs, the expression of antithrombogenic gene HSPG and endothelial NOS was determined using real-time PCR. Results are presented in Fig. 10. Results demonstrated significantly higher expression of HSPG of HUVECs than that of WJ-MSCs and AM-MSCs (*P* < 0.001). Interestingly, the expression of HSPG of WJ-MSCs was significantly higher than that of AM-MSCs (*P* < 0.001). There was no significant statistical difference of NOS expression between HUVECs, WJ-MSCs, and AM-MSCs (*P* > 0.05).

### Effects of cell sheet formation on cell viability

**Fig. 11:**
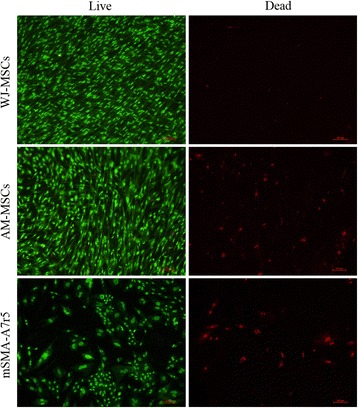
Representative fluorescence microscopic images of Live/Dead analysis of WJ-MSC- and AM-MSC-derived cell sheets and mSMC-A7r5. Live cells were stained *green* and dead cells were stained *red*. WJ-MSCs and AM-MSCs were primarily alive and dead cells were relatively few. Scale bar, 100 μm. *AM-MSCs* amniotic membrane mesenchymal stem cells, *WJ-MSCs* Wharton’s jelly mesenchymal stem cells

To obtain functional substitutes and provide more insights into the viability differences of WJ-MSCs, AM-MSCs, and mSMA-A7r5, the Live/Dead assessment was performed. The live cells stained green and the dead cells stained red (Fig. 11). WJ-MSCs sheets exhibited the more superior activities than the AM-MSCs sheets and mSMA-A7r5 demonstrated the worst activities. This data indicated that WJ-MSCs were a suitable cell source for cardiovascular regeneration and the construction of CTE grafts.

## Discussion

This work highlights the potential of the WJ-MSCs to produce CTE constructs as a suitable cell source. The cardiovascular system has a low regenerative capability, which makes the development of tissue engineering the practicable alternative in cardiovascular tissue regeneration. To construct a functional CTE substitute, a suitable seeding cell is needed, which acts as the functional substitute for autologous cells. It must be rapidly expanded in vitro to obtain sufficient numbers for basic research as well as clinical applications. Moreover, ideal seeding cell should be accessible, isolated, be capable of rapid expansion in in vitro culture, have immunology and hematology compatibility, and be capable of long-term survival and integration. In the field of cell-based CTE and regenerative medicine, there are two seeding-cell models typically pursued. Initially, CTE involved the isolation and expansion of autologous cells from vascular donor tissues [30]. Recently, a variety of tissue-derived MSCs have provided a viable cell source for future regeneration of cardiovascular tissues, resulting from their inherent ability of self-renewal and rapid proliferation [31]. Perinatal annexes can be collected without invasive procedures and ethical constraints. They provide an inexhaustible source of MSCs. In particular, perinatal annex-derived MSCs are a predominantly suitable source of MSCs, which are maintained in the embryological phase and retain some of the primitive stemness properties, and possess superior naivety and plasticity than adult tissue-derived MSCs and could be better suited for cardiovascular regeneration [7, 32, 33]. Furthermore, umbilical cord Wharton’s jelly and amniotic membrane provide a preferable tissue source of MSCs resulting from their easier accessibility and availability for homotransplantation. These inherent advantages make WJ-MSCs and AM-MSCs suitable sources for CTE and further studies should be performed. Thus, the data of the present study revealed that WJ-MSCs were a desirable seeding cell for CTE, which yielded superior advantages of cell proliferation, antiplatelet adhesion, hemocompatibility and viability than AM-MSCs. Moreover, WJ-MSCs demonstrated similar ECM deposition capacity to AM-MSCs during cell sheet preparation.

In the design of our experiments, WJ-MSCs and AM-MSCs isolation and characterization from perinatal annexes were achieved first. To compare MSCs derived from umbilical cord Wharton’s jelly and amniotic membrane, the proliferation and self-renewal capacity, the expression of specific surface antigens, and the multipotent differentiation potential were characterized according to the minimal criteria for MSCs recommended by the International Society of Cell Therapy (ISCT) [34]. Results demonstrated that both WJ-MSCs and AM-MSCs exhibited a similar morphology (Fig. 1a), immunophenotype (Fig. 2), and trilineage differentiation capacities (Fig. 3), and both met the definition established for MSCs by the ISCT. Furthermore, flow cytometry analysis showed that both WJ-MSCs and AM-MSCs partially expressed CD146 and α-SMA (Fig. 2). CD146 is one of the markers of pericytes [35, 36] and α-SMA is one of the differentiation markers expressed by vascular SMC [37]. Pericyte and SMC are the two major cell types of blood vessels. The presence of CD146 and α-SMA indicated that both WJ-MSCs and AM-MSCs populations are suitable for the development of tunica media and adventitia for tissue engineering vascular grafts.

Interestingly, this study demonstrated that the proliferation and self-renewal capacity of WJ-MSCs was significantly higher than AM-MSCs (*P* < 0.001). As shown in Fig. 1, WJ-MSCs consistently grow faster and have a higher frequency of CFU-F than AM-MSCs. WJ-MSCs have a rapid population doubling time and superior self-renewal capability, and this property could overcome the limitations of the slow growth rate with mature somatic cells, such as SMC and EC, the most popular seeding cells for CTE. These results suggest that WJ-MSCs can easily fulfill the need of clinical scale with a higher expansion capacity than AM-MSCs. The superior proliferative and self-renewal potential of WJ-MSCs may be associated with the higher expression of telomerase and stemness gene [38–40]. Hsieh et al. demonstrated that the gene expression involved in the phosphoinositide 3-kinase (PI3K)-AKT survival/proliferation pathway of WJ-MSCs was higher than that of bone marrow MSCs (BM-MSCs) [32]. Besides, several studies have demonstrated that WJ-MSCs with a low immunogenicity did not express the costimulatory complex (e.g., CD40, CD80, and CD86) and HLA-DR on their surface and characterized by broad immunomodulation properties [41–43]. These studies revealed that WJ-MSCs would be tolerated in allogenic transplantation.

The healthy EC is inherently nonthrombogenic and plays the essential role in regulating thrombosis and vascular tone. When CTE grafts are implantated and come into contact with blood, a number of thrombotic events occur, which correlate to CTE prostheses exposed to blood. To prevent thrombosis and activation of the coagulation cascade, EC had been seeded on biomaterials before implantation in a number of applications [24, 44, 45]. Therefore, HUVECs were used as a positive control in this study for investigation of the antiplatelet adhesion and hemocompatibility properties of WJ-MSCs and AM-MSCs.

In the construction of CTE grafts for clinical application, the achievement and maintenance of a confluent antiplatelet surface in the lumen is being pursued to improve blood compatibility and host integration and EC is widely accepted as an exclusively seeding cell. However, autologous EC has a limited proliferative capacity due to the age and disease status of the patient and the long time course of EC isolation and cultivation [46, 47]. Regarded as a functional substitute for EC, a number of seeding cells have been investigated for the construction of CTE grafts, such as endothelial progenitor cells (EPCs) [48, 49], BM-MSCs [50, 51], induced pluripotent stem cells (iPS) [52], and WJ-MSCs-transdifferentiated EC [53].

The results of this study are the first confirming the particular properties of WJ-MSCs of antiplatelet adhesion and nonactivation of the intrinsic pathway in vitro. SEM evaluation demonstrated that no aggregated platelets were found on the surface of WJ-MSCs-based cell sheets, and it was similar to HUVECs. However, aggregated platelets were visualized on the surface of AM-MSCs-based cell sheets (Fig. 4f). In addition, the quantification analysis of platelets demonstrated significant reduction (*P* < 0.001) in AM-MSCs- and non-cell-seeded groups compared to HUVECs- and WJ-MSCs-seeded groups as well as normal blood sample (Fig. 4g). Similar to this study, Zhao et al. showed that BM-MSCs had similar antiplatelet adhesion properties to EC [54]. Furthermore, Hashi et al. also revealed the unique antithrombogenic property of BM-MSCs due to the expression of HSPG on the BM-MSC surface [50].

Generally, blood coagulation is described in two phases, wherein primary platelets aggregate formation followed by the activation of the coagulation cascade to form the fibrin clot. In the blood coagulation cascade, thrombogenesis is initiated by the activation of the extrinsic or intrinsic pathway [44]. The PT and APTT tests are widely used in clinical diagnostic detection of abnormality in the extrinsic and intrinsic pathways, respectively [55, 56]. The shortening of either time indicates the activation and the prolongation of either time indicates a deficiency or inhibition of the respective cascade. In this work, the APTT of AM-MSCs was significantly shortened and HUVECs was significantly prolonged compared to that of normal blood sample and WJ-MSCs (Fig. 5b). In this study, real-time PCR analysis showed that WJ-MSCs had higher expression of HSPG than AM-MSCs (Fig. 10), which may be the important mechanism contributing to the WJ-MSCs’ resistance to platelet adhesion, and had superior hemocompatibility. These data demonstrated that WJ-MSCs had superior hemocompatiblity than AM-MSCs, including antiplatelet adhesion and avoidance of the activation of intrinsic coagulation pathway. These traits make WJ-MSCs an ideal cell source to construct CTE grafts.

Besides hemocompatibility requirements, the deposition of new ECM by the seeding cell is another critical challenge for CTE graft remodeling and regeneration as well as assembling into a functional substitute. Moreover, secretion of ECM by seeding cell is crucial to prevent the degeneration of the graft structure [7, 57]. Three-dimensional tissues reconstructed using the self-assembly approach of cell sheets represent excellent tools to explore the properties of ECM deposition and remodeling in vitro and the ECM is deposited and organized endogenously by the cells. Consequently, this study investigated the ECM secretion properties of WJ-MSCs and AM-MSCs based on scaffoldless cell sheet engineering. The cell sheet is an effective approach to explore ECM deposition. In a seminal article by L’Heureux et al., tissue engineering blood vessels were successfully produced by combining the ECM production nature of vascular SMC and fibroblasts and the hemocompatibility of EC [58]. However, this source of SMC, fibroblast, and EC are not compatible with a fully homologous approach. SMC from elderly persons have limited proliferative capability and a reduction in collagen production, which impair the application of CTE, especially in engineered vascular grafts [59]. To fulfill the expectancy of ECM deposition, it would be preferable to seek another potential source of SMC and fibroblasts. The results of this study revealed that both WJ-MSCs and AM-MSCs possess the property of ECM deposition. To the best of the authors’ knowledge, these results initially revealed that WJ-MSCs hold superior properties of collagen deposition and high expression of connexin-43, vimentin, laminin, as well as HSPG; however, AM-MSCs have preferable capability of elastin secretion and high expression of vitronectin and fibronectin. In a previous study, Huang et al. demonstrated that BM-MSCs sheets retained endogenous extracellular matrices and improved post-infarcted cardiac function after transplanted in a porcine model [60]. Furthermore, data from Au et al. suggested that BM-MSCs are perivascular cell precursors and functioned as perivascular cells for ECM deposition and BM-MSCs may serve as seeding cells for construction of tissue engineering vascular graft [61]. In consideration of the ECM assembly capacity of adipose stem cell, adipose stem cell-derived cell sheets have been successfully used in the treatment of myocardial infarction [62] and production of tissue-engineered vascular grafts [63]. These studies demonstrated that adult MSCs have the properties of ECM deposition and hold extensive application prospects and feasibility in CTE. In view of their properties of ECM secretion and cell sheet formation, we think that both WJ-MSCs and AM-MSCs are suitable for myocardial regeneration.

Another important parameter in MSCs-based clinical application is the cell survival and retention posttransplantation at the injury sites. In clinical settings, the resulting living MSCs reflect the biological properties of the terminal CTE grafts and cell sheet-based regenerative medicine. In this report, the cell viability profiles of WJ-MSCs, AM-MSCs, and mSMA-A7r5 were investigated by using Live/Dead cell viability assessment (Fig. 11). The results revealed that WJ-MSCs hold the highest viability after cultivation 14 day in vitro.

This study has some potential limitations. Importantly, this is an in vitro study to describe the potential of WJ-MSCs and AM-MSCs in CTE and cardiovascular regeneration. Although there are promising results with WJ-MSCs, in vivo studies are required to evaluate the regenerative and clinical translational properties of WJ-MSCs, such as construction-engineered blood vessels and myocardial infarction therapy. Moreover, further experiments will be needed to ascertain the exact molecular mechanism of WJ-MSCs antiplatelet adhesion and the other biological properties of WJ-MSCs as a substitute of EC, such as regulation of systemic blood flow, vascular tone, vascular inflammation and others. These further evaluations may accelerate the use of WJ-MSCs in clinical practice from bench to bedside.

## Conclusions

In summary, data from this study demonstrated that a positive role of WJ-MSCs to behave as endothelial and interstitial cells, which could act as a functional cell source for CTE and clinical cardiovascular regeneration. WJ-MSCs possess preferable proliferation capability and comparable properties of antiplatelet adhesion and did not activate the coagulation cascade to EC. However, platelet aggregation was visualized on the surface of AM-MSCs-derived cell sheets and the intrinsic pathway was activated. Furthermore, WJ-MSCs have superior properties of collagen deposition and higher viability than AM-MSCs during cell sheet formation. Taken together, WJ-MCSs could serve as an appealing and practical single-cell source for cardiovascular tissue engineering and regenerative therapy.

## Abbreviations

AM-MSCs: Amniotic membrane mesenchymal stem cells
APTT: Activated partial thromboplastin time
BM-MSCs: Bone marrow mesenchymal stem cells
CFU-F: Colony-forming unit fibroblast
CTE: Cardiovascular tissue engineering
EC: Endothelial cell
ECM: Extracellular matrix
FBS: Fetal bovine serum
HSPG: Heparin sulfate proteoglycan
HUVECs: Human umbilical vein endothelial cells
MSCs: Mesenchymal stem cells
MTT: 3-(4, 5-dimethylthiazol-2-yl)-2,5-diphenyltetrazolium bromide
NOS: Nitric oxide synthase
PBS: Phosphate-buffered saline
PRP: Platelet-rich plasma
PT: Prothrombin time
SEM: Scanning electron microscope
SMC: Smooth muscle cell
TEM: Transmission electron microscopy
WJ-MSCs: Wharton’s jelly mesenchymal stem cells
α-MEM: α-Minimum essential medium
α-SMA: α-smooth muscle actin
